# Altered functional connectivity is related to impaired cognition in left unilateral asymptomatic carotid artery stenosis patients

**DOI:** 10.1186/s12883-021-02385-4

**Published:** 2021-09-13

**Authors:** Shihao He, Ran Duan, Ziqi Liu, Cai Zhang, Tian Li, Yanchang Wei, Ning Ma, Rong Wang

**Affiliations:** 1 Department of Neurosurgery, Beijing Tiantan Hospital, Capital Medical University, Beijing, 10070 China; 2 Department of Neurosurgery, Peking University International Hospital, Beijing, 102206 China; 3 Collaborative Innovation Center of Assessment for Basic Education Quality, Beijing Normal University, Beijing, 100875 China; 4 Center of Stroke, Beijing Institute for Brain Disorders, Beijing, 100069 China

## Abstract

**Background:**

Asymptomatic carotid artery stenosis (aCAS) impairs haemodynamic and cognitive functions; however, the relationship between these changes and brain network connectivity remains largely unknown. This study aimed to determine the relationship between functional connectivity and neurocognition in patients with aCAS.

**Methods:**

We compared functional status in 14 patients with aCAS and 15 healthy controls using resting state functional magnetic resonance imaging sequences. The subjects underwent a full range of neuropsychological tests and a graphical theoretical analysis of their brain networks.

**Results:**

Compared with controls, patients with aCAS showed significant decline in neuropsychological functions, particularly short-term memory (word-memory, *p* = .046 and picture-memory, *p* = .014). Brain network connectivity was lower in patients with aCAS than in the controls, and the decline of functional connectivity in aCAS patients was mainly concentrated in the left and right inferior frontal gyri, temporal lobe, left cingulate gyrus, and hippocampus. Decreased connectivity between various brain regions was significantly correlated with impaired short-term memory. Patients with aCAS showed cognitive impairment independent of known vascular risk factors for vascular cognitive impairment. The cognitive defects were mainly manifested in the short-term memory of words and pictures.

**Conclusions:**

This study is the first of its kind to identify an association between disruption of functional connections in left carotid stenosis and impairment of short-term memory. The findings suggest that alterations in network connectivity may be an essential mechanism underlying cognitive decline in aCAS patients.

**Clinical trial registration-URL:**

Unique identifier: 04/06/2019, ChiCTR1900023610.

**Supplementary Information:**

The online version contains supplementary material available at 10.1186/s12883-021-02385-4.

## Background

Asymptomatic carotid artery stenosis (aCAS) is characterized by extracranial internal carotid artery stenosis in the ipsilateral carotid region, usually without a history of stroke or transient ischemic attack (TIA) [1]. Increasing evidence suggests that cognitive impairment may occur in not just symptomatic patients with a history of cerebral infarction or other cerebrovascular events, but also in those with aCAS [2]. Further, it has been shown that severe internal carotid artery stenosis (> 50%) is associated with a higher incidence of silent cerebral infarction [3]. In the mouse model of chronic cerebral insufficiency, although the mice did not develop motor dysfunction, their spatial learning ability through the maze test was reduced and they developed varying degrees of cognitive impairment [4]. These findings suggest that aCAS may not, in fact, be asymptomatic.

At present, functional neuroimaging is often used to reveal brain activity, which has become an important tool in cognitive research [5]. Brain network graph analysis provides an intuitive and attractive framework for studying the functional connections of brain information transmission and its correlation with behaviors [6], which may be used to explain some of the cognitive deficits in other diseases [7, 8]. However, few studies have investigated whether changes in the brain network are associated with altered cognitive function in patients with severe aCAS. Although cerebral hypoperfusion or infarction may be the cause of severe aCAS, the pathophysiological mechanism of brain function and network connectivity changes is still unclear [9–12].

This study aimed to determine the association between altered brain network connectivity and cognitive impairment in patients with aCAS using a graph theoretical analysis. We compared brain activities and cognitive function between 14 patients with severe aCAS and 15 matched healthy controls using neuropsychological tests and resting-state functional magnetic resonance imaging (rs-fMRI) scans.

## Materials and methods

### Participants

The participants in this prospective study consisted of 14 patients with unilateral aCAS who attended our institution between December 2018 and July 2019. After patients’ baseline data were collated and analysed, 15 healthy volunteers were recruited based on age, sex, and years of education.

### Inclusion and exclusion criteria

Inclusion criteria were as follows: [1] age 53–76 years; [2] left carotid artery stenosis ≥70% and right carotid artery stenosis < 50%; [3] right-hand dominance; [4] no history of stroke, TIA, dementia, and depression; [5] an education level of primary school or above; [6] The mini-mental state exam (MMSE) score was between 27 and 30;

The exclusion criteria were as follows: [1] posterior circulation stroke/stroke syndrome; [2] neuropsychiatric diseases (e.g., Alzheimer’s disease or Parkinson’s disease), severe systemic diseases, or a history of stroke; [3] contraindications of MRI scans (e.g., metal implants); [4] take medications that may affect cognitive function; (5) hunger or fatigue; or [6] Cannot complete the test task independently.

### Neuropsychological assessments and MRI data processing methods

For the neurocognitive function test, we adopted the method of He et al. [13] Specific cognitive tests are detailed in the supplementary materials. The neuropsychologists, who had no knowledge of each patient’s clinical data, used a computer workstation to test the participants. Neuropsychological examination and functional magnetic resonance examination are separated by less than 5 days. In the correlation analysis of the connections between brain regions, the network-based-*statistical analysis* was first used to conduct a statistical test on the edge linkage of the two groups. NBS analysis and correction were carried out for the connecting edges based on the level of the mass. For the first time, 0.001 was used to retain all the connecting edges with *P* value less than 0.001, and NBS correction was carried out for the remaining connecting edges with *P* value card 0.05. Detailed MRI parameters and fMRI data processing procedures are shown in the online supplementary materials.

### Statistical analyses

Neuropsychological data were analysed using the Statistical Package for Social Sciences (IBM SPSS version 20.0 for Windows), and *p* < 0.05 was considered statistically significant. For cognitive analysis, the control group was matched with the patient group in terms of age, sex, education, and hand dominance. Analysis of variance was conducted to exclude the influence of sex and risk factors on cognition performances. A two-sample t-test was performed to analyse differences between the patients and controls. All values are presented as mean ± standard deviation. All participants’ cognitive scores were correlated with significant brain network attributes using Pearson correlation analysis. The results for the model with statistically significant differences were also adjusted for multiple comparisons using the false discovery rate (Benjamini-Hochberg procedure).

## Results

In order to reduce the limitations of multiple factors on the study results, 14 patients with unilateral aCAS (average age, 63.6 years) were included in this study through strict inclusion criteria, and 15 healthy controls (average age, 62.4 years) were matched according to age, sex, years of education, and underlying diseases. No significant difference in sex, educational status, and other clinical factors were observed between the two groups (Table 1). The carotid ultrasound diagnosis of the included patients was severe left carotid stenosis marked 70–99%. There were no patients with significant unilateral occlusion.

**Table 1:** Basic characteristics of study participants

Characteristics	Patients (*n* = 14)	Controls (*n* = 15)	*p-* Values
Age (years)	63.6 (6.246)	62.4 (5.804)	.584
Sex			
Male: Female	9:5	10:5	>.99
Education (years)	9.86 (2.742)	9.00 (2.535)	.389
Risk factors (%)			
Hypertension	11 (78.6)	8 (53.3)	.245
Diabetes mellitus	6 (42.9)	5 (33.3)	.710
Ischaemic heart disease	3 (21.4)	2 (13.3)	.651
Hypercholesterolemia	5 (35.7)	4 (26.7)	.700
Smoking	6 (42.9)	6 (40)	>.99

Values are shown as the number of cases (%) unless otherwise indicated.

The patients showed significantly poor performances on the neuropsychological tests, especially the short-term memory test for Chinese words (*p* = .046), and the picture memory test (*p* = .014). Raven’s Standard Progressive Matrices (*p* = .063), executive function (*p* = .058), and choice RT (*p* = .053) were low in the patient group, but the differences were not significant (Table 2).

**Table 2:** Neuropsychological assessments

	Patients, n = 14	Controls, n = 15	
Variables	Mean (SD)	Mean (SD)	*p-*values
CRT_RT	1159.36 (902.439)	641.80 (172.601)	.053
CRT_ACC	97.00 (6.288)	99.20 (1.424)	.221
SPM	11.71 (5.757)	15.53 (4.853)	.063
ROT	9.57 (5.893)	13.53 ((7.190)	.118
VWM1	6.07 (1.817)	6.73 (1.438)	.285
VWM2	4.36 (1.336)	4.80 (1.699)	.444
SUB	29.64 (9.145)	34.40 (7.189)	.130
COMSUB	13.29 (5.239)	13.07 (6.193)	.919
WORDM	47.57 (16.195)	58.80 (12.667)	.046*
PICTM	66.29 (7.878)	73.07 (5.994)	.014*
EXCUT1	0.57 (3.228)	1.47 (2.924)	.440
EXCUT2	0.07 (3.912)	−2.73 (3.731)	.058

Abbreviations: SD, standard deviation; CRT_RT/ACC, Choice reaction time_ reaction time/ accuracy; SPM, Raven’s Standard Progressive Matrices; ROT, Mental rotation; VWM, verbal working memory, digit span, 1, Recite in order, 2, Recite in reverse order; SUB, Simple subtraction; COMSUB, Complex subtraction; WORDM, word-memory; PICTM, picture-memory; EXCUT, Executive function, 1, same direction, 2, Opposite direction. *p < .05

Values are shown as the number (percentage) of cases unless otherwise indicated. The mean values are presented with the standard deviation. SD, standard deviation.

**Fig. 1:**
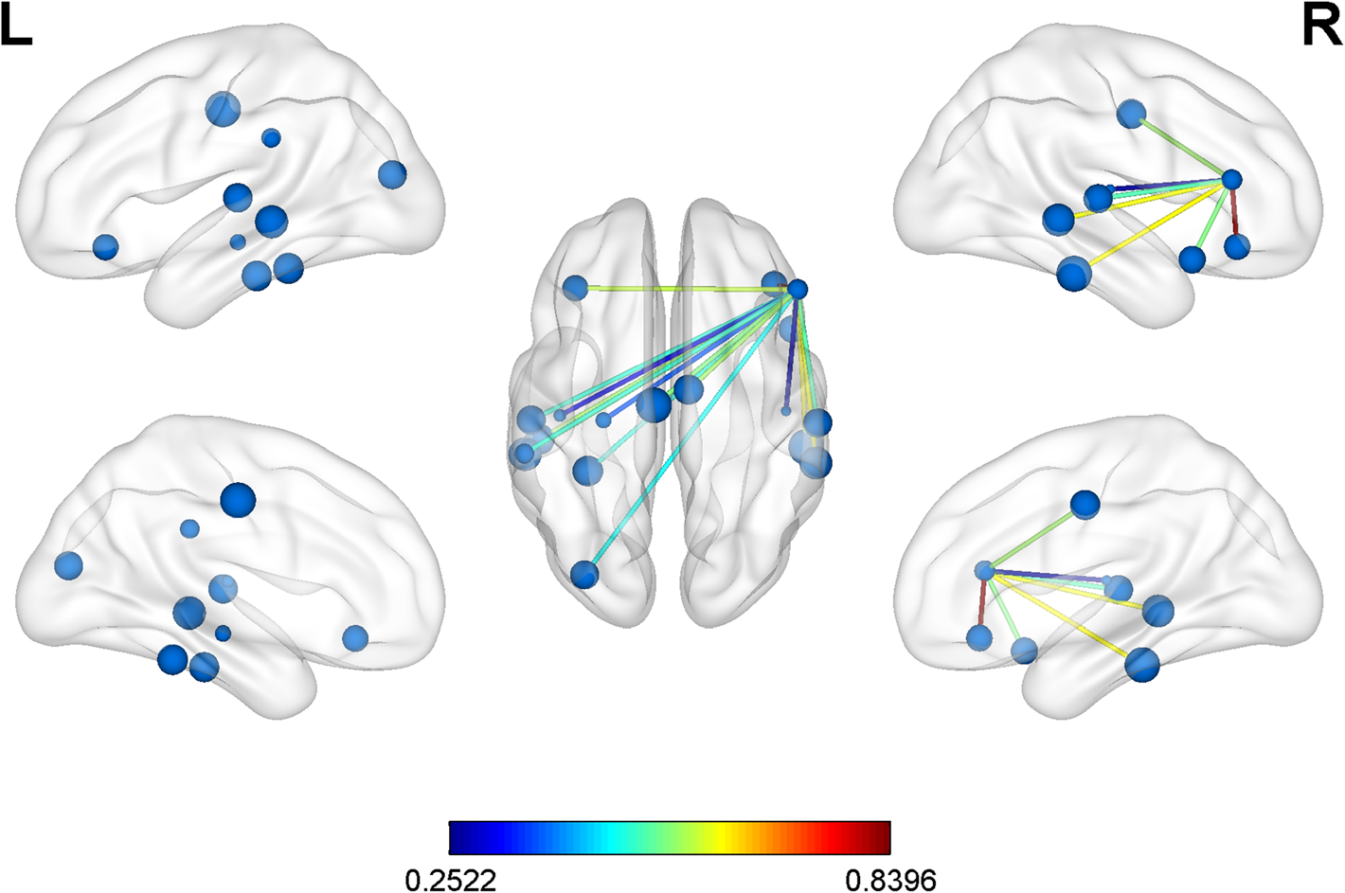
**Different connections between patients and healthy controls.** The nodes and edges shown in the figure were significantly lower in patients than in healthy controls (aCAS < HC). The size of the node is the degree value; The range of colorbar represents the strength of a functional connection, from 0.25 to 0.83 aCAS, asymptomatic carotid artery stenosis; HC, healthy controls.

In the graph theory analysis, there were no significant differences between the patients and the healthy controls for the node-based network attributes and global parameters after the false discovery rate (FDR) test (Table 3), including assortativity (*p* = .057), hierarchy (*p* = .080), network efficiency (*p* = .596), small-worldness (*p* = .372), or synchronisation (*p* = .881). The small-world attribute had a significant correlation with short-term word memory in the aCAS group (r = −.634, *p* = .015). The functional connections between the inferior frontal gyrus of the right hemisphere and the left orbital inferior frontal gyrus, hippocampus, superior temporal gyrus, and middle temporal gyrus were significantly low in the aCAS patients compared with the controls (Fig. 1).

**Table 3:** Differences in global attributes between the two study groups

	Patients, *n* = 14 Mean (SD)	Controls, *n* = 15 Mean (SD)	*t*-value	*p*-value
Assortativity	3.4142 ± 0.8961	2.8113 ± 0.7369	1.985	0.057
Hierarchy	−0.1791 ± 0.3812	0.0408 ± 0.2633	−1.819	0.080
Network efficiency	0.2008 ± 0.0093	0.2024 ± 0.0069	−0.537	0.596
Synchronization	−0.6175 ± 0.6950	−0.5814 ± 0.5907	− 0.151	0.881
Small-worldness	0.3927 ± 0.0626	0.4141 ± 0.0642	−0.908	0.372

The aCAS patients showed a significantly lower edge connection than the healthy controls *(p* = .0316). A reduction in the edge property of the triangular part of the right inferior frontal gyrus (AAL label No. 14) connected to the other 17 nodes was observed.

**Fig. 2:**
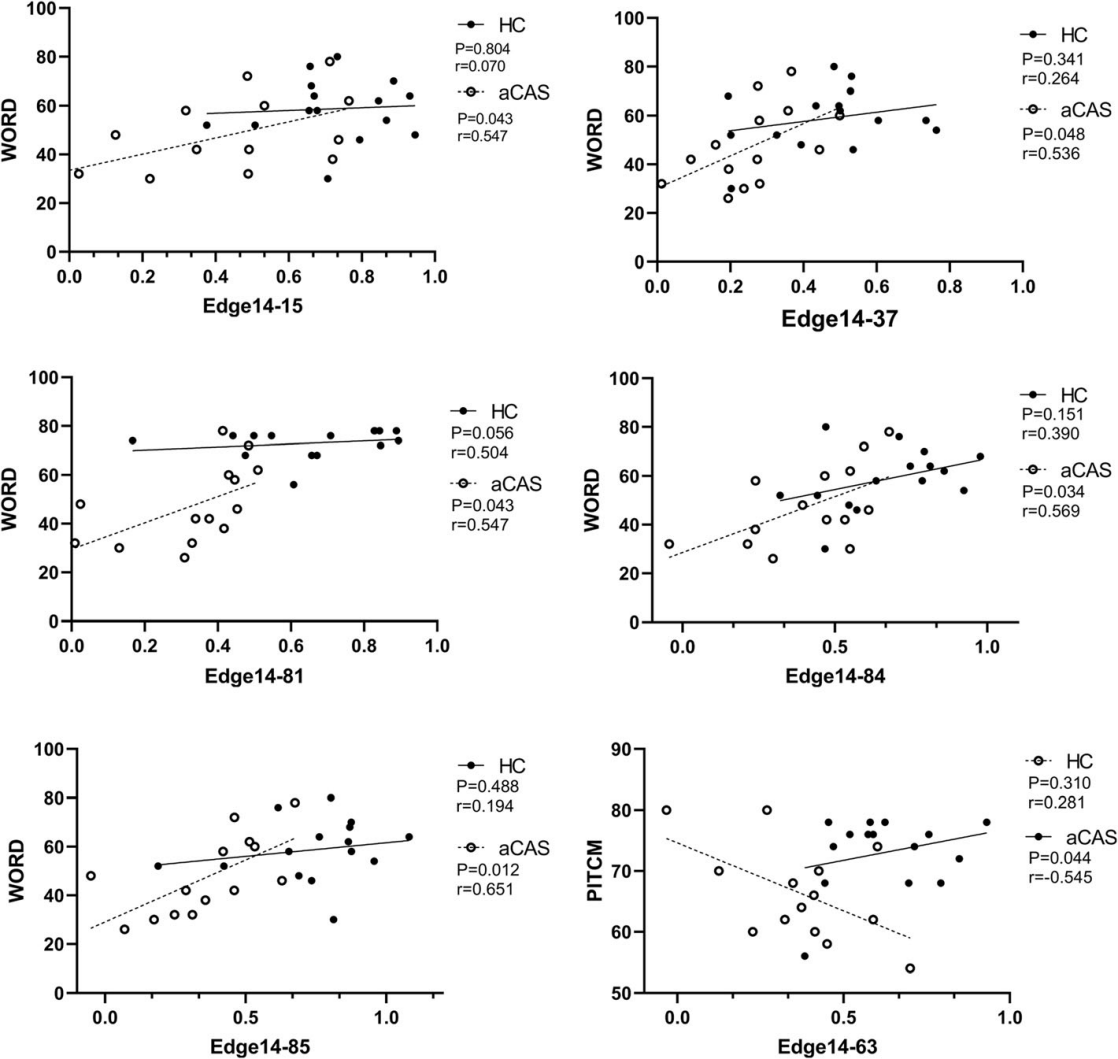
**Correlations between WORDM and PICM and connections**. Six scatter charts for correlation analysis of edge attributes with short-term memory scores of words and pictures are shown. The regression lines were calculated in two groups. All probability values were adjusted by FDR correction. WORDM, word-memory; PICM, picture-memory; HC, healthy controls; aCAS, asymptomatic carotid artery stenosis; FDR, false discovery rate. Edge14: IFGtriang. R, Inferior frontal gyrus, triangular part; Edge15: ORBinf. L, Inferior frontal gyrus, orbital part; Edge37: HIP.L,Hippocampus; Edge81: STG. L, Superior temporal gyrus; Edge84: TPOsup. R, Temporal pole: superior temporal gyrus; Edge85: MTG. L, Middle temporal gyrus; Edge63: SMG. L, Supramarginal gyrus

As shown in Fig. 2, functional connections between the right triangular inferior frontal gyrus and the left orbital inferior frontal gyrus, the left hippocampus, the right superior temporal gyrus and the left middle temporal gyrus (the AAL brain regions of 14–15, 14–37, 14–84, and 14–85) were all correlated with word short-term memory score, with positive correlation in the patient group and negative correlation in the control group. In the correlation analysis, functional connections between the the right triangular inferior frontal gyrus and the left superior temporal gyrus (the AAL brain regions of 14–81) were positively correlated with word short-term memory in the patient group and the control group.

Additionally, in the correlation analysis of functional connections between the right triangular inferior frontal gyrus and the Superior left margin (the AAL brain regions of 14–63) were all correlated with picture short-term memory score, with negative correlation in the patient group and positive correlation in the control group.

**Fig. 3:**
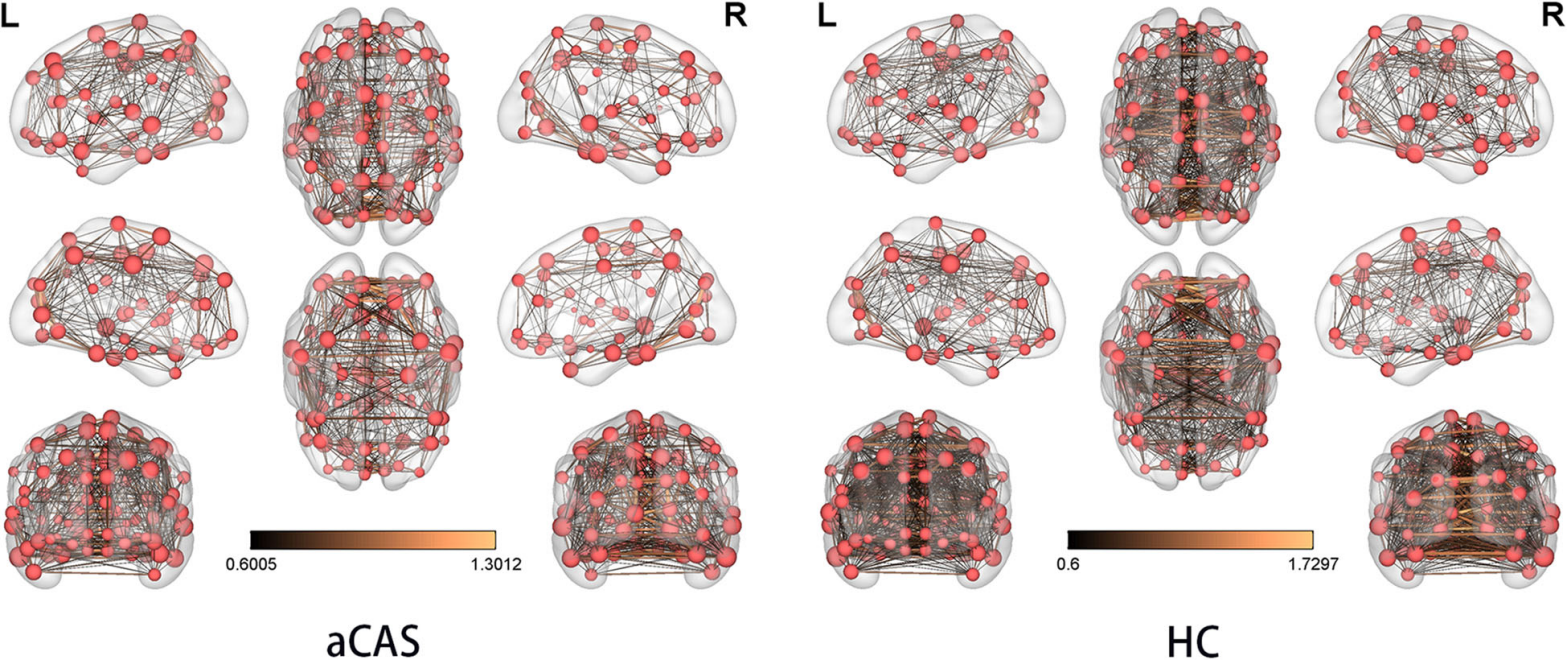
**Brain connectivity maps.** Brain connectivity maps of patients with aCAS are shown on the left, and those of healthy controls are shown on the right (Threshold = .60, density = .20). aCAS, asymptomatic carotid artery stenosis

As shown in Fig. 3, compared with the control group, functional connections related to brain network information transmission were more sparse in the patient group.

## Discussion

Using rs-fMRI, this prospective study comprehensively elucidated the alterations in brain functional connectivity in patients with aCAS, and determined the correlation between altered brain functional connectivity and cognitive impairment. Our results suggest that short-term memory and other cognitive functions decline in aCAS patients prior to the onset of clinical symptoms, and that changes in brain network connectivity may be associated with cognitive impairment.

A previous study observed that patients with aCAS gave a significantly poor performance on the MMSE and the Montreal Cognitive Assessment [14]. However, in our previous studies, we only observed minor changes in orientation, recall ability of three things, and language ability in aCAS patients. Therefore, we conducted a more detailed and systematic test using computer workstations to determine the specific extent of cognitive impairment in aCAS patients who had normal MMSE scores.

Cognitive impairment seems to selectively affect mental ability in patients, and some studies have demonstrated that stroke affects cognitive functions [10, 12, 15]. This study found a statistically significant decline in short-term word and picture memory ability in aCAS patients, but not in reasoning and judgment ability, executive function, and reaction speed. Notably, calculation ability and spatial imagination ability were well-preserved in these patients. Some pictures are easier to remember than words, and educational status may have some effects on the short-term memory of images and words. Additionally, studies have also demonstrated that decrease in image memory becomes less pronounced over time [16, 17]. Therefore, we further analysed the short-term memory decline after excluding the effects of age and education. We found that the decline in short-term memory in aCAS patients was still significant after adjusting for age and education. Therefore, the ability of short-term memory of words might be predominantly impaired in these asymptomatic patients. Numeric-span task was associated with short-term memory to some extent. Although the results of this task showed a decrease in the patient group, no statistically significant difference was found (*p* = .285), which may be not related to the sample size of this study. The number - span task was often associated with executive function. The functional areas of short-term memory of numbers and words should be further studied.

One study found that the amplitude of low-frequency fluctuation values of the bilateral dorsomedial prefrontal cingulate cortex, praecuneus, superior temporal gyrus, and inferior parietal lobule decreased significantly in patients with aCAS, indicating a significant decrease in neural activity [18]. In this study, we used rs-fMRI data to construct the brain functional network to analyse the changes in network connectivity. Our study suggests that rs-fMRI may be used to detect an early decline in brain connections and predict the state of patients’ memory.

A study found a markedly decreased blood oxygenation level-dependent correlation between opposite hemispheres when the ROI was predefined on the stenotic side (flipped to the left) in aCAS patients, suggesting a disruption of interhemispheric connectivity in these patients; [9] however, this finding is controversial because there are some differences between the left and right sides of the brain. Our graph theory analysis showed reduced connectivity between the brain hemispheres in aCAS patients, and global efficiency was not significantly different between the aCAS patients and the healthy controls. These findings are similar to those observed in patients with symptomatic carotid artery stenosis, indicating that cerebral blood supply has a strong compensatory effect in chronic disease course [19]. It is thought that the brain of patients with aCAS could reconcile a good deal of short-range connections for segregation, while maintaining a sufficient number of remote connections to ensure processing integration. In our previous hemispheric network study, we found that the communication of CAS patients’ coordination and information on the affected side of the brain was significantly impaired, but the contralateral brain network has shown the brain network compensation effect. In the left cerebral hemisphere of patients with CAO and CAS of the right carotid artery, connections between the Rolandic operculum and insula, median cingulate and paracingulate gyri and postcentral gyrus, and superior parietal gyrus and precuneus were stronger than those in normal controls [20]. Therefore, the results suggest that patients with carotid artery stenosis have different effects on the affected side, and that hemispheric template results may be more accurate for the global analysis of graph theory. In addition, the brain network connection will be reduced in the affected side and compensated in the healthy side.

There was no significant difference in node attributes of the graph theory analysis after FDR correction between the patient and control groups. However, the functional edge property was significantly lower in aCAS patients than in the controls (*p* = .0316). Additionally, a reduced efficiency of functional connections in brain networks was observed in patients with aCAS (Fig. 3). In asymptomatic patients, the brain network is damaged and the node response is not significant, which may be slightly related to the illness, but also represents a higher sensitivity of functional connections.

The decline of functional connectivity in aCAS patients was mainly concentrated in the left and right inferior frontal gyri (IFG), temporal lobe, left cingulate gyrus, and hippocampus (Fig. 1). Our findings suggest that the weak association between these areas and the triangular part of the right inferior frontal gyrus may be directly related to short-term memory decline in patients. We further found that five functional connections between the triangular part of the inferior frontal gyrus (AAL no.14) and other brain regions were significantly correlated with short-term memory decline. Previous studies have suggested that IFG may be associated with affective empathy [21], and abnormalities in this functional connection have also been found in patients with depression [22]. At the same time, it has been pointed out that the two clusters in the posterior extra frontal flow (IFG) are functionally related to inhibition and implementation, while the two preclusters are related to reasoning and social cognition processes [23]. This area may be involved in different functions, and we found that reduced connections between this area and other brain areas may be associated with decreased short-term memory. This finding may suggest that cognitive function may be dominated not only by a single brain region or node, but also by multiple regions or networks.

The correlations between short-term word memory and the left orbital inferior frontal gyrus, left hippocampus, right superior temporal gyrus, left middle temporal gyrus, or right frontal lobe were reduced in aCAS patients, compared with healthy controls. At present, the function of the temporal lobe is not understood fully, but the sense of hearing is one of its main functions. It has been suggested that some visual resolution, as well as memory, language and some motor functions, may depend on the temporal lobe [24] [25]. Notably, increasing evidence suggests that the hippocampus may be associated with short-term memory [26], and damage on both sides of the hippocampus may cause short-term memory loss, without affecting long-term memory. Therefore, the reduced brain network connections between the temporal lobe, hippocampus, and the triangular part of the right inferior frontal gyrus might be an important mechanism responsible for the short-term word-memory decline. These correlations were not significant in normal controls. It may also be related to insufficient samples in this study, and we will continue to conduct longitudinal studies.

Recent studies have shown that the left supramarginal gyrus is one of the key nodes of the short-term memory network involved in retaining an abstract representation of serial order information, independently from the content information [27]. The decline of functional connections between the right inferior frontal gyrus and the left supramarginal gyrus is related to the impairment of short-term memory (r = −.545, *p* = .044). This finding suggests that connections in these brain regions may indeed be important for short-term memory.

The purpose of this study was to increase the understanding of the mechanism of cognitive impairment by determining the correlation between cognitive impairment in aCAS and functional connectivity of brain networks, and to help define the criteria for intervention in the treatment of the disease. This study has some limitations. In order to reduce the limitation of multi-factors on the study results, we only included patients with unilateral left carotid artery stenosis in this study, and we intend to conduct a longitudinal study on patients with right carotid artery stenosis in the future. Additionally, the type of cognitive impairment may differ according to hand-dominance in the patients. Therefore, further studies including more patients with different hand-dominance and stenosis are needed to confirm our current findings.

## Conclusions

We observed that the short-term memory of patients with left aCAS was highly correlated with an altered brain network connection. The alterations in network connections may be an important mechanism underlying the decline of cognitive function in patients with aCAS. Further, our findings indicate that rs-fMRI may be used to identify an early decline in brain connections and predict the state of patients’ memory through correlation analysis of the brain network in various brain regions.

## Supplementary Information

**Additional file 1 SUPPLEMENTARY MATERIALS.** The online supplementary materials include the introduction of the cognitive function test scale involved in this study as well as the detailed parameters of fMRI data, data pre-processing process, functional network analysis method and all the references involved.

## Data Availability

All data generated or analyzed during this study are included in the published article. Some or all data, models, or code generated or used during the study are available from the corresponding author by request.
