# Supplementary material for: Altered functional connectivity is related to impaired cognition in left unilateral asymptomatic carotid artery stenosis patients
Source: BMC Neurol. 2021 Sep 13;21:350. doi: 10.1186/s12883-021-02385-4 (PMC8436468; doi:10.1186/s12883-021-02385-4)
Supplement: Supplementary file 1 — Additional file 1 SUPPLEMENTARY MATERIALS. The online supplementary materials include the introduction of the cognitive function test scale involved in this study as well as the detailed parameters of fMRI data, data pre-processing process, functional network analysis method and all the references involved. [file 12883_2021_2385_MOESM1_ESM.pdf]

Altered Functional Connectivity is Related to Impaired Cognition in Unilateral Asymptomatic Carotid Artery Stenosis Patients

SUPPLEMENTARY MATERIALS

Shihao He,[1,*] Ran Duan,[3,*] Ziqi Liu,[1] Cai Zhang,[4] Tian Li,[4] Yanchang Wei,[1] Ning Ma,[1] Rong Wang[1, 2, 3]

[1]Department of Neurosurgery, Beijing Tiantan Hospital, Capital Medical University, Beijing 10070, China

[2]Center of Stroke, Beijing Institute for Brain Disorders, Beijing 100069, China

[3]Department of Neurosurgery, Peking University International Hospital, Beijing 102206, China

[4]State Key Laboratory of Cognitive Neuroscience and Learning & IDG/Mc Govern Institute for Brain Research, Beijing Normal University, Beijing 100875, China

[*]SH and RD contributed equally to this work.

Correspondence to:

Rong Wang, MD

E-mail: ronger090614@ccmu.edu.cn

The supplementary materials include the introduction of the cognitive function test scale involved in this study as well as the detailed parameters of fMRI data, data pre-processing process, functional network analysis method and all the references involved.

Neuropsychological assessments

The choice reaction time (RT) task, which was adapted from the simple RT task from Butterworth's Dyscalculia Screener (2003),(1) was used to assess the manual response effect and main processing speed.

All cognitive assessments were performed using the Online Psychological Experimental System. Each task includes a practice session and a formal testing session. All tasks have shown acceptable half-split reliability values of 0.80–0.96 in previous studies.(2-4)

Nonverbal matrix reasoning was used to assess general intelligence and abstract reasoning ability, which has been correlated with mathematical performance.(5, 6) The task was adapted from Raven's standard progressive matrices.(7) Each question has 4–6 potential responses and only one answer is correct. Participants were asked to identify the missing image from a sequence according to the rules behind it.

Mental rotation was used to evaluate visuospatial ability. The test was adapted from the study by Vandenberg and Kuse.(8) The revised version had only two choices and was limited to 3 min. Each trial had three three-dimensional images, one shown at the top of the screen and the other two at the bottom. Participants were asked to judge which of the two candidates at the bottom was the same as the top one after mentally rotating one of the images. The correct image was rotated from the original, with a rotation angle ranging from 15° to 345° (at 15°-intervals). The other image was a mirror image of the target. Participants pressed the "Q" key to select the image on the left side or "P" to select the image on the right side. An adjusted number of correct trials was used (see the sentence completion test).

A verbal working memory test was used to measure working memory capacity. The digit span test from the Wechsler intelligence scale was used. The test was divided into two parts, a forward digit span task representing short-term memory and general attention and a backward digit span task representing working memory related to executive function.(9, 10)

Simple subtraction was used to assess simple calculation ability and magnitude representation.(4) The task involved 92 subtraction problems with a correct single-digit answer. For each trial, a subtraction question (for example, 8-3) appeared at the top of the screen and two candidate answers at the bottom. The minuend of each question ranged from 2 to 18 and the answers ranged from 2 to 9. The official test time limit was 4 minutes.

The complex subtraction task had 95 problems, with each problem involving double-digit numbers for both operands. Most problems required mathematical borrowing. In each trial, a subtraction problem (e.g., 63–27) was presented at the top of the screen, with two candidate answers at the bottom. The difference between the true and false answers was 1 or 10. Formal testing of the task was limited to 3 min.

Word-memory ability and visual short-term memory were measured using the short-term memory span for Chinese words and phrases and the picture short-term memory test, respectively.(11) During the learning stage, a series of words and pictures was presented on the screen. In the test, the subjects judged whether the test images had been presented in the learning phase. If so, they pressed the "Q" key using their left index finger; otherwise, they pressed the "P" key with their right index finger. A multistep, multilocation search task was used to evaluate executive function.(12)

The Edinburgh Handedness Inventory was used to investigate left and right handedness.[18] The subjects filled in the Edinburgh handedness questionnaire, where a final score ≥4 was classified as right handedness and scores ≤-4 were classified as left handedness; intermediate scores were classified as double handedness.(13)

The participants were tested by neuropsychologists, who were blinded to each patient's clinical data, using computer workstations. The interval between neuropsychological testing and MRI examination was <5 days.

MRI acquisition

MRI data were obtained using a 3.0-Tesla MR system (Verio A Tim+Dot System, Siemens, Germany). A standard 12-channel head coil (3T Head MATRIX, A Tim Coil, Siemens) was used for signal reception. Each participant lay supine with their head snugly secured by a belt and foam pads. During rs-fMRI scans, participants were asked to close their eyes but not fall asleep, and to not think about anything in particular. The scanning parameters were as follows: repetition time, 2220 ms; echo time, 30.0 ms; voxel size, 3.0 × 3.0 × 3.0 mm; field-of-view, 192 mm; slice thickness, 3.0 mm; 32 slices; and total scanning time, 9 min 11 s.

Data pre-processing

Rs-fMRI data pre-processing was conducted using GRETNA software (http://www.nitrc.org/projects/gretna/)(14) in MATLAB (Matlab Release 2013b, Mathworks Inc., Natick, MA). The first six volumes of individual functional images were discarded to allow for magnetisation equilibrium. A slice-timing correction was implemented to align the rs-fMRI images to the middle slice. Individual images were realigned (standard of removal: 3 mm) so that each part of the brain was in the same position in every volume, and they were then warped into the standard Montreal Neurological Institute (MNI) space by applying the transformation matrix, which can be derived by registering the T1 weighted image (co-registered with functional images) into the MNI template using unified segmentation. Smoothing (4 × 4 × 4 mm) was used to improve the signal-to-noise ratio and attenuate anatomical variances caused by inaccurate inter-subject registration after spatial normalisation. Nuisance signals were removed from each voxel's time series to reduce the effects of non-neuronal fluctuations (including head motion profiles, and cerebrospinal fluid and white matter

signals) and linear trends in the signals. Rs-fMRI data were band-pass filtered to reduce the effects of low-frequency drift and high-frequency physiological noise. Regions of interest (ROI) were placed using the Anatomical Automatic Labelling (AAL) atlas. Pearson's correlations for all time-course pairs were computed for each participant and transformed into z-scores via Fisher's transformation.

Functional network analysis

The following steps were performed for functional connectivity matrix construction and analyses using GRETNA software. GRETNA contains parcellation schemes that are defined by randomly parcelling the brain into 1,024 ROIs. Pre-processed rs-fMRI images were structurally defined into the AAL-90 atlas. The mean time series was extracted from each parcellation unit, and pairwise functional connectivity was estimated among the time series by calculating linear Pearson's correlation coefficients (r). After calculating Pearson's r between each pair of 90 ROIs, a 90 × 90 correlation matrix was constructed for each participant.

For the network analysis, various topological properties of a network were calculated from both global and nodal characteristics, which can be compared with random network counterparts to determine non-randomness.

For the graph theory analysis, six node-based and three global parameters were obtained for each network. The node-based network parameters included nodal-clustering coefficient (C), shortest path length, nodal efficiency, local efficiency (Eloc), degree centrality (DC), and betweenness centrality (BC); the global parameters included global efficiency (Eglob), small-worldness (s), and assortativity coefficient (A). Finally, we calculated the area under the receiver operating characteristic curve (AUROC) for each network metric. Mathematical definitions of these parameters have been described elsewhere.(15)

The significant regions and the corresponding AAL region numbers

AAL No.	Abreviation	Brian region
14	IFGtriang.R	Inferior frontal gyrus, triangular part;
15	ORBinf.L	Inferior frontal gyrus, orbital part
37	HIP.L	Hippocampus
63	SMG.L	Supramarginal gyrus
81	STG.L,	Superior temporal gyrus
84	TPOsup.R	Temporal pole: superior temporal gyrus
85	MTG.L	Middle temporal gyrus

Correlations between word-memory and connections

No.	CAS-*r*	CAS-*P*	HC-*r*	HC-*P*
edge14-15	.547*	.043	.070	.804
edge14-37	.536*	.048	.264	.341
edge14-81	.547*	.043	.504	.056
edge14-84	.569*	.034	.390	.151
edge14-85	.651*	.012	.194	.488

Correlations between picture-memory and connections

No.	CAS-*r*	CAS-*P*	HC-*r*	HC-*P*
edge14-63	-.545*	.044	.281	.310

References

1. B. B. Dyscalculia screener. NferNelson Pub, . 2003.
2. Wei W, Lu H, Zhao H, Chen C, Dong Q, Zhou X. Gender Differences in Children's Arithmetic Performance Are Accounted for by Gender Differences in Language Abilities. Psychological Science. 2012;23(3):320-30.
3. Wei W, Yuan H, Chen C, Zhou X. Cognitive correlates of performance in advanced mathematics. Br J Educ Psychol. 2012;82(Pt 1):157-81.
4. Zhou X, Wei W, Zhang Y, Cui J, Chen C. Visual perception can account for the close relation between numerosity processing and computational fluency. Front Psychol. 2015;6:1364.
5. Minna Kyttälä JEL. Some factors underlying mathematical performance: The role of visuospatial working memory and non-verbal intelligence. European Journal of Psychology of Education. 2008;23:77-94.
6. Rohde TE, Thompson LA. Predicting academic achievement with cognitive ability. Intelligence. 2007;35(1):83-92.
7. Raven J. The Raven's Progressive Matrices: Change and Stability over Culture and Time. Cognitive Psychology 2000;41:1-48.
8. Kuse SgVaAr. Mental rotations, a group test of three-dimensional spatial visualization. Perceptual and Motor Skills.47:599-604.
9. Heather M. Conklin BS, Clayton E. Curtis, Ph.D., Joanna Katsanis, Ph.D., and William G. Iacono, Ph.D. Verbal Working Memory Impairment in Schizophrenia Patients and Their First-Degree Relatives: Evidence From the Digit Span Task. Am J Psychiatry. 2000;157:275–7.
10. LaBelle DR, Lee BG, Miller JB. Dissociation of Executive and Attentional Elements of the Digit Span Task in a Population of Older Adults: A Latent Class Analysis. Assessment. 2017;26(7):1386-98.
11. Yu Bolin JQ. Stm span for chinese words and phrases. Acta Psychologica Sinica.

1985;17:25-32.
12. Zelazo PD, Reznick JS, Spinazzola J. Representational flexibility and response control in a multistep multilocation search task. Developmental Psychology. 1998;34(2):203-14.
13. Dellatolas G, De Agostini M, Curt F, Kremin H, Letierce A, Maccario J, et al. Manual skill, hand skill asymmetry, and cognitive performances in young children. Laterality. 2003;8(4):317-38.
14. Wang J, Wang X, Xia M, Liao X, Evans A, He Y. GRETNA: a graph theoretical network analysis toolbox for imaging connectomics. Front Hum Neurosci. 2015;9:386.
15. Rubinov M, Sporns O. Complex network measures of brain connectivity: uses and interpretations. Neuroimage. 2010;52(3):1059-69.
